# The Lancet

**Published:** 1885-02-20

**Authors:** 


### THE LANCET.

We extract the following from an interesting private letter received from Dr. E. W. Lane, of Georgia:

In editor's note to the article copied from Ther. Gazette in Jan. No. of RECORD, "Shall we Bleed," I see the outcroppings of an honest heart. The lancet, as well as many other good remedies which were formerly used by the profession, has been driven out (too much) to make room for the more fashionable remedies of the present day. There are many in the profession who have heen practicing medicine several years that have never performed venesection, nor seen it done. That the lancet has been abused, none will pretend to deny; but that is no just reason why it should not be used judiciously when indicated.

